# LC-MS/MS based detection of circulating proinsulin derived peptides in patients with altered pancreatic beta cell function

**DOI:** 10.1016/j.jchromb.2022.123482

**Published:** 2022-10-05

**Authors:** Rachel E Foreman, Claire L Meek, Geoffrey P Roberts, Amy L George, Frank Reimann, Fiona M Gribble, Richard G Kay

**Affiliations:** 1 Wellcome-MRC Institute of Metabolic Science-Metabolic Research Laboratories, Level 4, Wellcome-MRC Institute of Metabolic Science, Addenbrooke’s Hospital, Cambridge, CB2 0QQ; 2 Peptidomics and Proteomics Core Facility, Level 4, Wellcome-MRC Institute of Metabolic Science, Addenbrooke’s Hospital, Cambridge, CB2 0QQ; 3 Department of Clinical Biochemistry/Wolfson Diabetes & Endocrine Clinic, Cambridge University Hospitals NHS Foundation Trust, Cambridge CB2 0QQ

**Keywords:** Liquid Chromatography Mass Spectrometry, Quantitation, Insulin, Clinical Analysis, Gastrectomy, Diabetes

## Abstract

Routine immunoassays for insulin and C-peptide have the potential to cross-react with partially processed proinsulin products, although in healthy patients these are present at such low levels that the interference is insignificant. Elevated concentrations of proinsulin and des-31,32 proinsulin arising from pathological conditions, or injected insulin analogues, however can cause significant assay interferences, complicating interpretation. Clinical diagnosis and management therefore sometimes require methods that can distinguish true insulin and C-peptide from partially processed proinsulin or injected insulin analogues. In this scenario, the high specificity of mass spectrometric analysis offers potential benefit for patient care.

A high throughput targeted LC-MS/MS method was developed as a fit for purpose investigation of insulin, insulin analogues, C-peptide and proinsulin processing intermediates in plasma samples from different patient groups. Using calibration standards and bovine insulin as an internal standard, absolute concentrations of insulin and C-peptide were quantified across a nominal human plasma postprandial range and correlated strongly with immunoassay-based measurements. The ability to distinguish between insulin, insulin analogues and proinsulin intermediates in a single extraction is an improvement over existing immunological based techniques, offering the advantage of exact identification of the species being measured. The method promises to aid in the detection of circulating peptides which have previously been overlooked but may interfere with standard insulin and C-peptide immunoassays.

## Introduction

**Figure 1:**
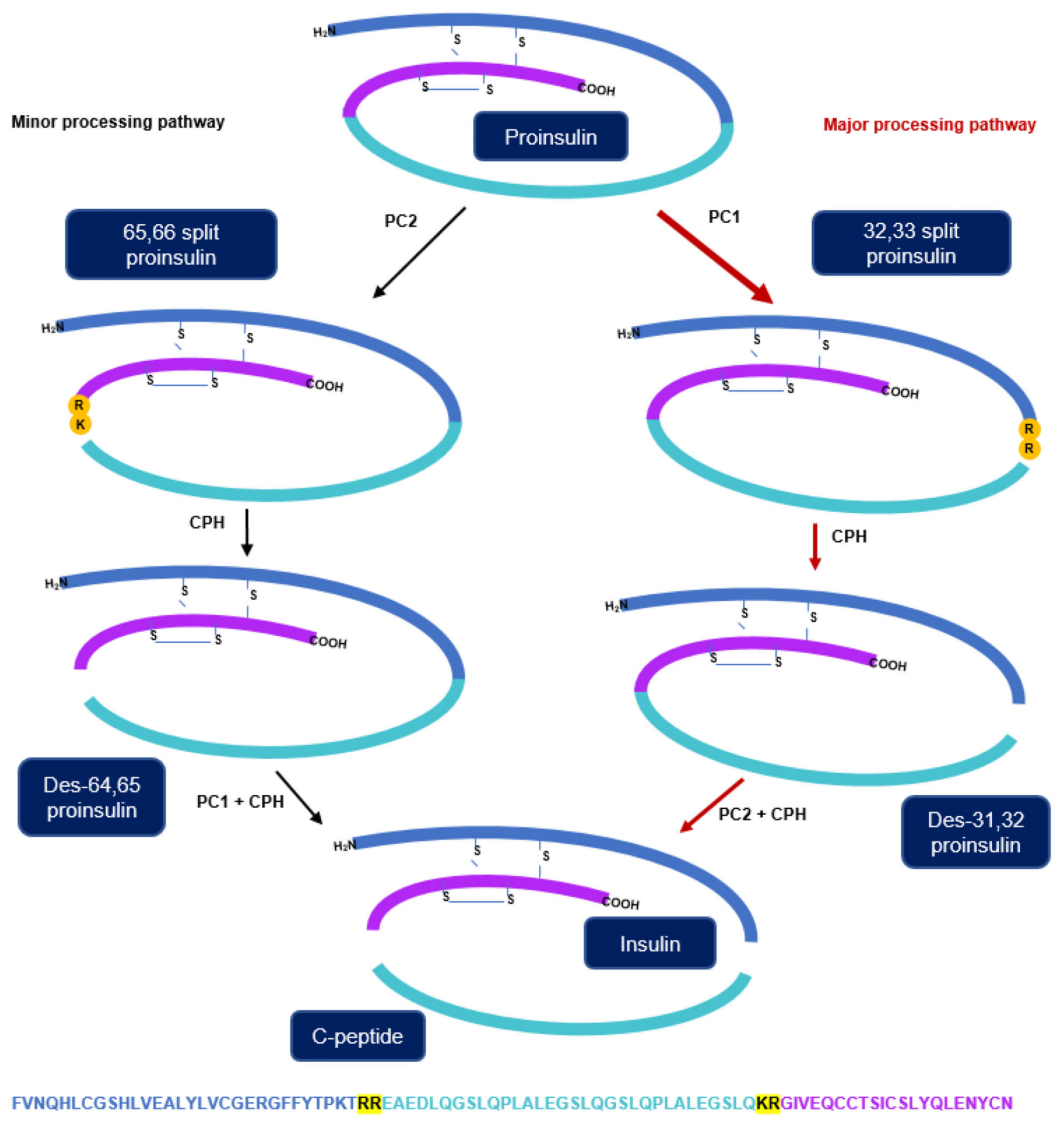
A schematic diagram of the processing pathway for the activation of insulin in pancreatic β cells. This process is mediated by a combination of Proprotein convertase 1 (PC1), Proprotein convertase 2 (PC2) and carboxypeptidase H (CPH). The amino acid sequence shown is for intact human proinsulin, which is made up of insulin A-chain (purple), B-chain (navy) and C-peptide (light blue), it is cleaved at the highlighted arginine and lysine residues

Insulin is a peptide hormone from pancreatic beta cells that helps to control the circulating blood concentration of glucose. It is biosynthesised as a prohormone (proinsulin) which requires processing into the mature peptide through multiple enzymatic cleavage steps (Figure 1). This processing involves prohormone convertases PC1/3, PC2 and carboxypeptidase H, and generates intermediary structures including 32-33 split proinsulin, des-31,32 proinsulin, 65-66 split proinsulin and des-64,65 proinsulin [1, 2].

Immunological based methods are typically used to measure insulin and C-peptide in plasma, as they are simple, cheap and effective. However, these methods may falter at the analysis of samples from complex patients as most antibodies to insulin and C-peptide can cross-react, to some extent, with proinsulin and partially processed proinsulin intermediates [3, 4] (Figure 1). Accurate measurements can also be affected by endogenous anti-insulin antibodies and the immunoassay kit antibodies cross reacting with insulin analogue medication [5]. In recent years, mass spectrometry has been developed as an alternative analytical technique for detecting insulin and correlates strongly with routine clinical assays [6]. It is especially useful when immunoassays generate conflicting data about different proinsulin derived products, such as in patients with Hirata’s disease who have auto-antibodies against insulin [7]. In these samples, specific extraction methodologies prior to LC-MS/MS analysis were used to disrupt antibody binding, allowing discrimination between free and antibody-bound insulin [8, 9].

Evidence of incomplete proinsulin processing has previously been detected in patients with type 2 diabetes (T2DM) [10–12], as well as people with rare polymorphisms in prohormone convertases [13, 14]. The bioactivity of proinsulin and des-31,32 proinsulin against the insulin receptor is only around 10% that of insulin [15] but as they have longer plasma half-lives, 92 min for proinsulin [16, 17] compared with the 4-6 min half-life of insulin [18], it is possible for them to accumulate in plasma to levels where they can have clinically significant glucose-lowering activity. Routine insulin assays are not able to discriminate between the PC1/3 cleavage product 32,33 split proinsulin [19] and the product of subsequent carboxypeptidase H processing – des-31,32 proinsulin [20]. These two peptides differ by only an arginine and lysine residue at the C-terminus of the A-chain, removed by carboxypeptidase H, which corresponds to a delta mass shift of 312 Da and is easily distinguishable by mass spectrometry [21].

Insulin analogue drugs have been revolutionary in the treatment of diabetes mellitus, but their high similarity to endogenous insulin can cause cross-reactivity with routine antibody-based insulin measurements [22], sometimes making it difficult to determine whether a raised level reported by an insulin assay reflects endogenous insulin secretion or an injected insulin analogue. A major benefit of using mass spectrometry is that it can distinguish between closely related insulin molecules, as it relies upon the assignment of mass to charge ratios (*m/z*), and can simultaneously quantify analogue drugs and endogenous insulin in plasma [23] and urine [24]. LC-MS/MS based methods can even differentiate between the isobaric LisPro (Humalog) insulin used to treat diabetes and endogenous insulin, due to a signature product ion derived from the transposition of the proline and lysine residue [25].

There are a number of published mass spectrometry-based methods for measuring insulin in clinical samples [26, 27], although few have included proinsulin and partially processed proinsulin species [28]. Those that have done so have used high resolution instrumentation[29, 30], which are rarely available in clinical assay laboratories and the much longer assay run times mean they are not suitable for fast turnaround clinical analysis.. Using a low resolution system, such as a triple quadrupole, may have a loss in sensitivity but these are able to monitor specific precursor/product ion pairs for multiple analytes with reasonable resolution. One issue with using targeted transitions is that the analyte can be overshadowed by high signals for later eluting plasma proteins with similar *m/z*. But this is easily overcome by optimisation of the liquid chromatography gradient, which allows for clear integration of the peptide of interest.

Here we describe a rapid LC-MS/MS method which targets multiple proinsulin derived peptides and insulin analogues in parallel, covering the nominal postprandial plasma insulin range[31]. Following standard bioanalysis guidelines[32] and using quality control samples this method was estabished as a fit for purpose method for the quantitation of endogenous insulin and c-peptide and four common insulin analogue drugs, with additional transitions for the detection of intermediate processing peptides. The method was developed on a triple quadrupole system, which can be found in many clinical laboratories, and was applied to plasma samples from human subjects with altered pancreatic beta cell function. Alongside healthy volunteers and people with T2DM, we studied a group with previous gastrectomy surgery who have increased post-prandial insulin secretion without known beta cell dysfunction, and pregnant women with and without gestational diabetes mellitus (GDM), in whom insulin requirements are increased as a result of pregnancy.

## Materials and method

### Chemicals

Unless stated otherwise all reagents were commercially sourced and used as supplied. HPLC grade methanol, acetonitrile, and water (Fisher Scientific, Loughborough, UK) were used for all analyses. Reagent grade bovine serum albumin, formic acid and acetic acid (Sigma Aldrich, Poole, UK) were used for extraction methods.

Reference standards for human insulin (Actrapid), C-peptide (Bachem), bovine insulin and four commonly prescribed insulin analogue drugs, Humalog (Lispro, Eli Lilly), Glargine (Lantus, Sanofi), Aspart (Novo Nordisk) and Detemir (Novo Nordisk) were stored at -20°C as 1 mg/mL solutions, in 20% methanol:0.1% formic acid: 0.1 % bovine serum albumin (BSA) (aq).

Human proinsulin standard 09/296 was purchased from NIBSC and only used for setting up *m/z* transitions and retention time.

EQAS (External Quality Assurance Services) quality control samples (BioRad) are routinely used by the Core Biochemical Assay Laboratory to validate their assays, and an insulin set were received on wet ice (approximately 4°C) for immediate extraction.

Human EDTA plasma taken after an overnight fast from a previous study [33] was pooled and used to prepare calibration and quality control (QC) samples.

In all analytical runs, the calibration standards were freshly prepared by serial dilution in human plasma starting with an intermediate 100 μg/mL solution (from a combined dilution of both insulin and C-peptide stock solutions). The QC samples were prepared independently, using a different intermediate solution but the same lot of pooled blank human plasma.

### Patients

Healthy volunteers and patients with T2DM were recruited to have a 75 g oral glucose tolerance test (OGTT) after an overnight fast. Details of the study and participants have been published previously [33]. The study was approved by the Research Ethics Committee (12/EE/0064) and all participants provided written informed consent. This was part of an intervention study to assess the effect of encapsulated nutrients or placebo upon gut hormone concentrations. Only data from the placebo visits were included in the current analysis. All patients with T2DM were free of diabetes complications, were treated with oral hypoglycaemic agents and had not previously received injectable insulin.

The study of gut hormones in gastrectomised patients and healthy control participants was reported previously [34]. This study was approved by the Research Ethics Committee (13/EE/0195 and 16/EE/0138) and all participants provided written informed consent. In brief, following an overnight fast, participants drank 50 g of glucose in 200 ml water and plasma samples were collected at timed intervals.

Pregnant women with risk factors for GDM were recruited from Cambridge University Hospitals NHS Foundation Trust antenatal clinics at around 28 weeks’ gestation. All women were asked to give an additional sample at the time of clinical antenatal blood sampling for routine diabetes testing. Due to the Covid-19 pandemic, an OGTT could not be performed given the requirement for social distancing. The study was ethically approved (20/EM/0133) and all participants provided written informed consent.

### Sample extraction and LC-MS/MS Analysis

Blood samples were collected into EDTA tubes, immediately placed on ice and centrifuged for 10 minutes at 3500 x *g* at 4°C. 400 μl plasma aliquots were snap frozen on dry ice and stored at -80°C.

For all calibration, QC and clinical samples a 100 μL plasma sample was precipitated with 500 μL 80% acetonitrile (aq), containing internal standard (2 ng/mL bovine insulin), and briefly vortex mixed. The samples were centrifuged at 4°C for 5 mins at 3500 x *g* and the supernatant was transferred to a clean 96-well QuanRecovery (Waters) plate. The supernatant was dried down under nitrogen at 40°C on a Biotage SPE Dry 96 Sample Concentrator System (Upsala, Sweden) and reconstituted in 200 μL 20% methanol, 0.1% formic acid (aq). The samples were extracted through an Oasis HLB PRiME μElution SPE plate (Waters), with 200 μL wash steps of 0.1% formic acid (aq) and 5% methanol, 1% acetic acid (aq), concurrently. The samples were eluted into a final QuanRecovery plate with 2 x 30 μL 60% methanol, 10% acetic acid (aq), diluted with 75 μL 0.1% formic acid (aq) and immediately injected onto the LC-MS system for analysis.

Targeted analysis of selected peptides was performed on a dual pump M-Class LC system with a trap valve manager (Waters) coupled to a Xevo TQ-XS mass spectrometer (Waters), with a 100 x 0.3 mm HSS T3 iKey (Waters). 10 μL of sample was injected onto the system (20 μL loop) and were initially trapped on a nanoEase M/Z Peptide BEH C18 Trap Column (130Å, 5 μm, 300 μm X 50 mm, Waters) at 15 μL/min for 3 minutes, with mobile phases set to 90% A (0.1% formic acid (aq)) and 10% B (0.1% formic acid (acetonitrile)). The iKey was set at 45°C and the analytes were separated over a 13-minute gradient from 10% to 55% B, at a flow rate of 3 μL/min. The column was flushed for 3 minutes at 85% B before returning to initial conditions, resulting in an overall run time of 20 minutes.

The mass spectrometer was set up in positive ion electrospray ionisation mode with a capillary voltage of 3 kV, a cone voltage of 30 V and source gas flow rate of 150 L/h. Collision energies were optimised for each peptide prior to sample analysis, with the collision gas set at a flow rate of 0.15 mL/min. The targeted SRM transitions (Table 1) were set up based on precursor and product ion fragments for each peptide. Each analyte was set to have a dwell time of 50 milliseconds.

Mass spectrometry data was processed on TargetLynx XS (version 4.2, Waters), quantitation and statistical analysis was performed using Microsoft Excel and GraphPad Prism 9.

## Results

### Establishing LC-MS/MS assays for insulin-related peptides

**Figure 2:**
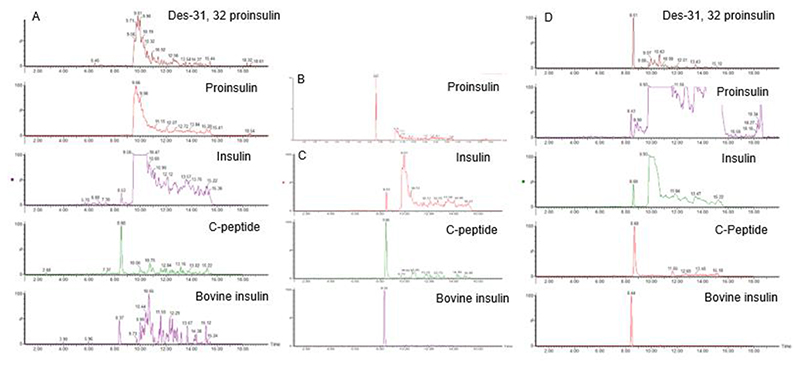
Targeted LC-MS/MS chromatograms A: Chromatograms for all monitored insulin peptide transitions in blank plasma used to prepare calibration standards and QCs. The endogenous concentration of insulin and C-peptide in this blank sample are 105 pg/mL and 1500 pg/mL respectfully, calculated using standard addition. B: Chromatogram for proinsulin reference solution (70 ng/mL), used to confirm retention time. C: Chromatograms for insulin and C-peptide spiked into human plasma in a 300 pg/mL calibration standard, together with bovine insulin which was added to samples as an internal standard (2 ng/mL). D: Targeted LC-MS/MS chromatograms for fully and partially processed insulin peptides in a plasma sample from a fasted type 2 diabetes patient, with bovine insulin internal standard. The quantified concentrations of insulin and C-peptide in this sample are 320 pg/mL and 2300 pg/mL respectfully.

Pooled human plasma samples were spiked with insulin and C-peptide to identify *m/z* and retention times (Table 1). Multiple transition pairs were assessed during development and establishment of the method, however the most suitable (best selectivity, best accuracy and most sensitive) were selected for the final method. Chromatograms for the selected ions in a calibration sample containing insulin, C-peptide and bovine insulin (internal standard) are shown in Figure 2C. Due to the limited availability of reference material for des-31,32 proinsulin and proinsulin, a positive control sample from a recent study [8] was used to determine their respective *m/z* values and retention times (Table 1), and for proinsulin this was confirmed with a proinsulin standard (Figure 2B).

To determine whether the different proinsulin-derived species would be detectable in native plasma, we examined a pilot series of plasma samples from healthy volunteers and people with T2DM. Peaks for endogenous insulin and C-peptide were identified in all samples, together with small peaks for proinsulin and des-31,32 proinsulin in some but not all samples (Figure 2D).

The LC-MS/MS method could detect and distinguish four clinically used insulin analogues when spiked into human plasma (Supplementary Figure 1). The cross interference of these peptides with endogenous insulin was assessed, and only Lispro was found to interfere with endogenous insulin analysis, but only at high Lispro concentrations. As these analogues were not detected in any patient samples, consistent with these participants not receiving exogenous insulin treatment, the potential contribution of Lispro to insulin measurement was not considered an issue in this data set. The *m/z* transitions and collision energies used to monitor insulin analogues are given in Table 1.

The m/z values for precursor and product ions, together with collision energies and retention times, for different products of proinsulin processing peptides and insulin analogues. Note, detemir elutes much later than the other insulins due to the fatty acid functional group attached. Values were obtained by spiking human plasma samples with exogenous standards, except for proinsulin and des-31,32 proinsulin which were obtained from a human sample with Hirata’s disease [8].

**Figure 3:**
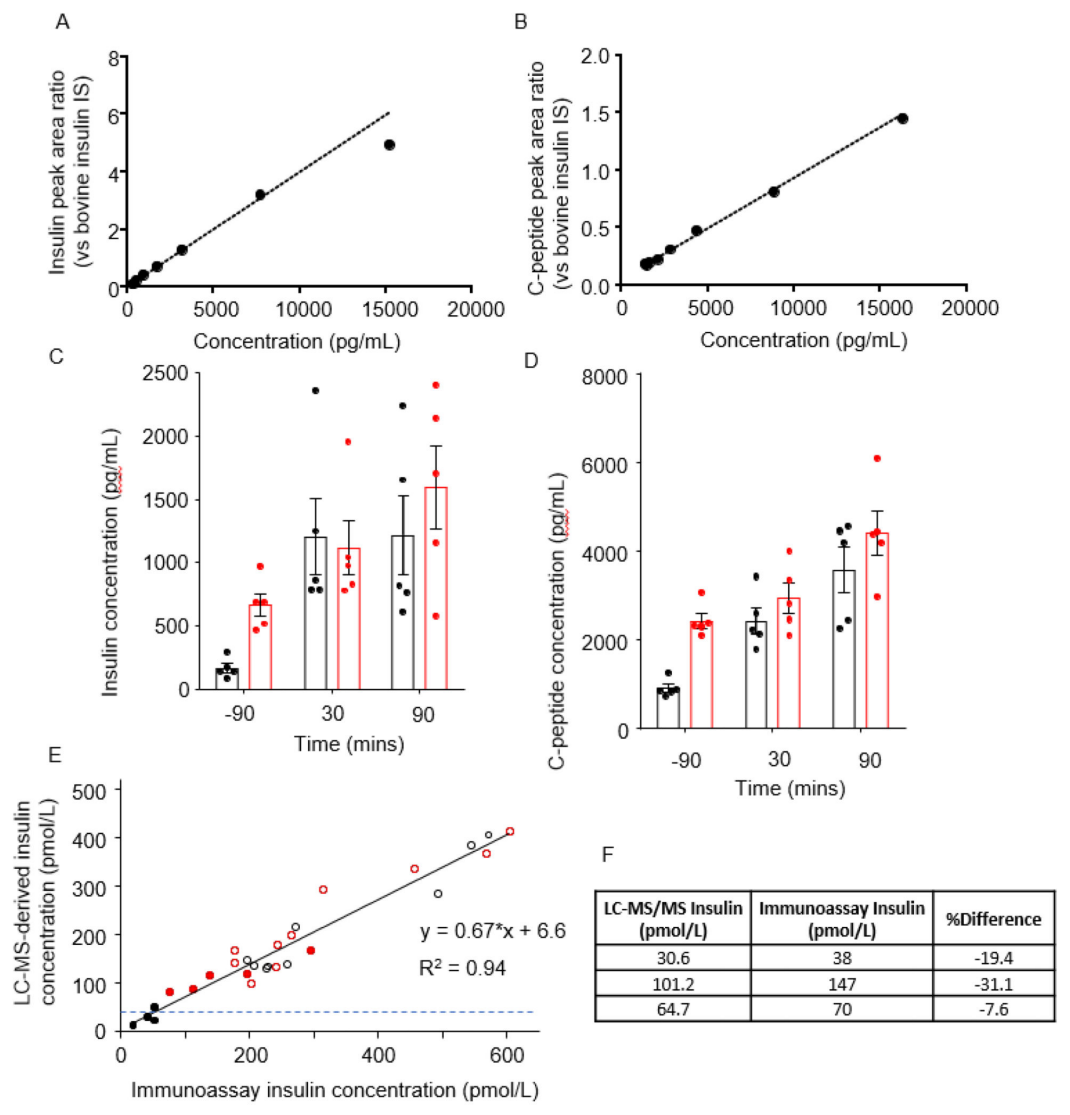
Insulin and C-peptide assays in patients with T2DM A, B. Calibration curves for insulin (A) and C-peptide (B), obtained by spiking pooled human plasma samples with exogenous insulin or C-peptide at different concentrations, fitted with a least squares (linear) 1/y2 weighted regression. The concentrations of the calibration standard samples have been corrected for endogenous concentrations in the control plasma matrix. C, D. Calibrated concentrations of insulin (C) and C-peptide (D), measured in plasma samples from healthy control (black) or type 2 diabetic (red) participants at the times indicated relative to consumption of a 75 g oral glucose tolerance test. Mean (±SEM error bars). E. Correlation between insulin values measured by LC-MS/MS compared with immunoassay in plasma samples from control (black) and T2DM (red) groups either fasting (filled symbols) or at different times after an oral glucose challenge (open symbols). The samples below the LLOQ limit of the LC-MS/MS method (dashed blue line) were found to be < 25% different from immunoassay values. Measured concentrations of three insulin EQAS samples, by both LC-MS/MS and immunoassay methods and the percentage difference between the results.

To enable quantification of insulin and C-peptide, we generated calibration standards by adding either insulin and C-peptide to pooled plasma from fasting individuals, over the concentration range 50 and 15,000 pg/mL (nominally 50, 100, 300, 750, 1,500, 3,000, 7,500, 15,000 pg/mL). All samples were additionally spiked with a bovine insulin internal standard during the extraction, enabling normalisation of the peak area of the test analyte to that of bovine insulin. Calibration curves for insulin and C-peptide were linear over this concentration range but did not pass through the origin due to endogenous insulin (approximately 100 pg/mL) and C-peptide (approximately 1500 pg/mL) present in the pooled fasted plasma used as the matrix (Figure 2A). We therefore took the “standard addition” approach of interpolating the linear fit back to the x-axis to estimate concentrations present in the plasma matrix, and generated corrected calibration curves (Figure 3A,B). Due to this, the lowest limit of quantitation was adjusted in each analytical run to the total peptide concentration in the lowest acceptable calibration standard. Any clinical sample results measured below this concentration were estimated using the linear regression and comparison to the immunoassay values showed % relative error < 25%.

The robustness of this approach was also validated by comparing the measured insulin responses to a clinical immunoassay in both patient samples and EQAS quality control samples (Figure 3E,F).

Quality control (QC) samples were analysed to determine the precision and accuracy of the LC-MS/MS method for insulin and C-peptide quantitation. Six QC samples were extracted at four levels and the mean measured concentrations were all within 30 % relative error to total (spiked and endogenous) peptide concentration (Tables 2 and 3).

Stability of insulin and c-peptide in QCs at sample storage (-70°C), and extraction (on ice) temperatures and following four freeze/thaw cycles was assessed during method development, by comparing stability QCs against a freshly prepared calibration curve. Both insulin and C-peptide were measured within acceptable relative error to prepared peptide concentrations in all storage conditions. The extraction recovery of both peptides was also assessed, as well as some dilution QCs up to three times the calibration range limit (nominally 45 ng/mL) (Supp Table 1).

Partial validation of the four insulin analogue drugs was also performed in QCs prepared in human plasma, to confirm the robustness of the assay. These were all measured within 25 % relative error and show that the assay is suitable for future samples from medicated patients (Supp Fig 2).

Quality control samples were prepared using pooled fasted plasma and n = 6 were analysed alongside a calibration curve to determine the robustness of the assay. Due to the presence of endogenous insulin, standard addition was applied to generate a corrected calibration curve and true QC values. The mean measured concentration values were used to calculate coefficient of variation (%CV) and relative error (%RE) for each QC level.

Quality control samples were prepared using pooled fasted plasma and n = 6 were analysed alongside a calibration curve to determine the robustness of the assay. Due to the presence of endogenous c-peptide, standard addition was applied to generate a corrected calibration curve and true QC values. The mean measured concentration values were used to calculate coefficient of variation (%CV) and relative error (%RE) for each QC level.

### Insulin and C-peptide in patient samples

We next applied the quantitative insulin and C-peptide method to plasma samples from a previous study of healthy control subjects and people with T2DM, taken following a 75 g oral glucose tolerance test, for which immunoassay-based insulin measurements were already published [33]. Insulin and C-peptide were quantifiable in all samples and their LC-MS/MS derived concentrations at different time points after glucose ingestion are shown (Figure 3C,D). Calibrated insulin concentrations calculated by LC-MS correlated well with those measured by immunoassay, including at concentrations that had been interpolated below that of the pooled plasma used to generate the standard addition curve, validating the use of this approach (Figures 3E).

**Figure 4:**
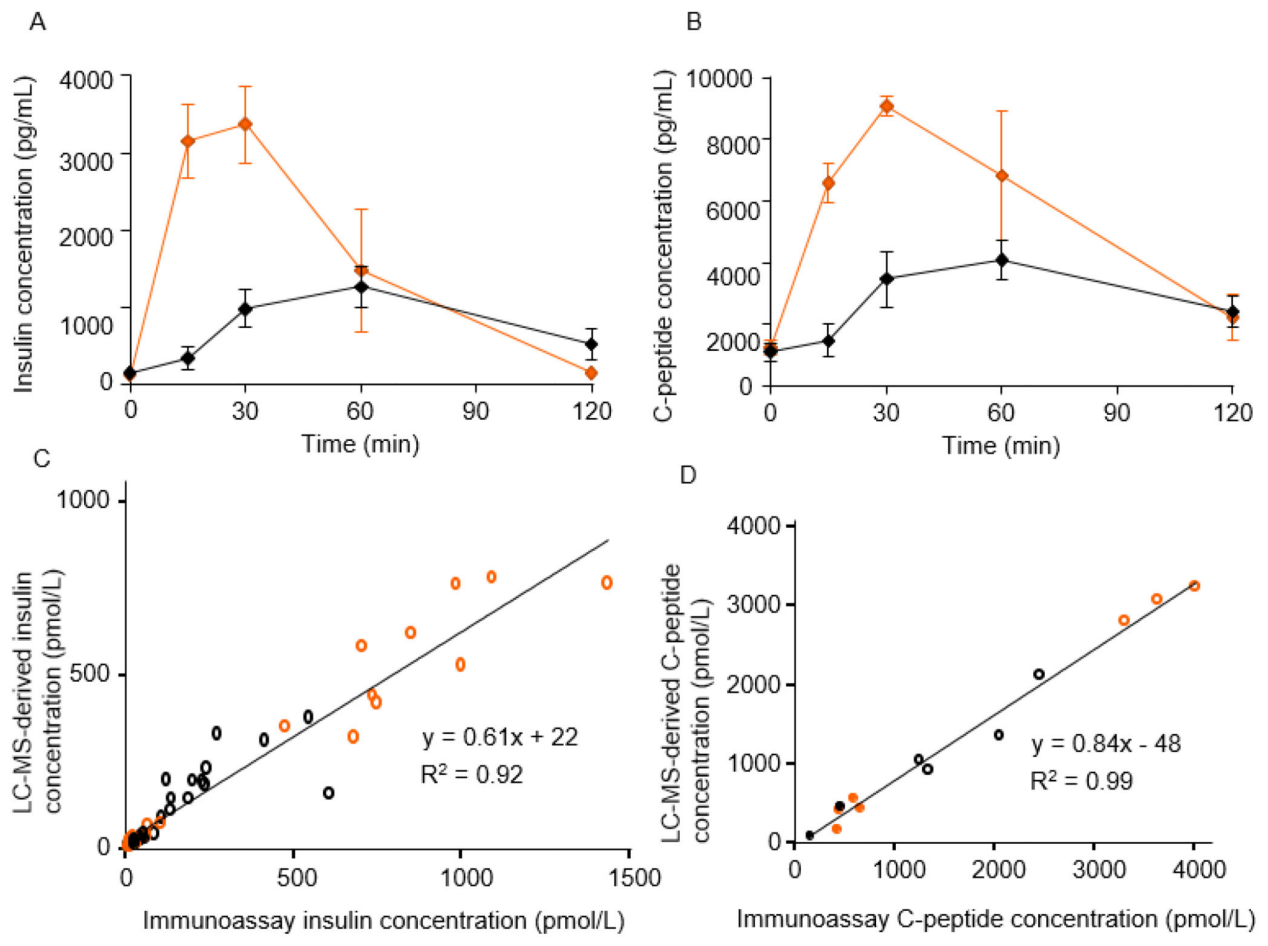
Proinsulin products in post-gastrectomy patients vs controls A,B. Mean (±SEM error bars) insulin (A) and C-peptide (B) concentrations, measured in plasma samples from healthy control (n=4-5, black symbols) and post-gastrectomy (n=3-4, orange) participants after a 50 g oral glucose tolerance test. C,D Correlation between insulin (C) and C-peptide (D) concentrations measured by LC-MS/MS compared with immunoassay in plasma samples from control (black) and post-gastrectomy (orange) participants either fasting (filled symbols) or at different times after an oral glucose challenge (open symbols).

To further test the LC-MS/MS assays, we quantified insulin and C-peptide in another cohort of five healthy controls and five patients post-gastrectomy, who had taken a 50 g oral glucose tolerance test as part of a previous study [34]. Consistent with previous immunoassay-based insulin measurements in these samples, the insulin concentrations determined by LC-MS/MS exhibited an earlier and elevated peak in the gastrectomy group compared with controls (Figure 4A), and C-peptide profiles followed a similar pattern, as expected (Figure 4B). C-peptide was additionally measured by immunoassay in a selection of these samples for comparison with the LC-MS approach. Strong correlations were observed between the immunoassay and LC-MS/MS generated concentrations for both insulin and C-peptide (Figure 4C,D). There was good agreement between the C-peptide plasma concentrations assigned using the two techniques (slope of 0.84, Figure 4D) however the agreement between immunoassay and LC-MS/MS derived plasma insulin concentrations was less convincing (slopes of 0.67, 0.61 in figure 3E and 4C respectively). An investigation into this discrepancy showed that the insulin standard used to generate the calibration line for the LC-MS/MS analysis was measuring high on the immunoassay (generating a negative bias of approximately -40% for the LC-MS/MS derived values as seen in the correlation plots). We believe this bias was due to an error in the preparation and/or storage of the standard used in the LC-MS analysis rather than a significant bias in the LC-MS approach, as earlier data showed tighter comparisons – see figure 3F). Furthermore, previous LC-MS analyses performed in our lab showed better agreements between techniques of 0.87 [9].

### Des-31,32 proinsulin in patient samples

**Figure 5:**
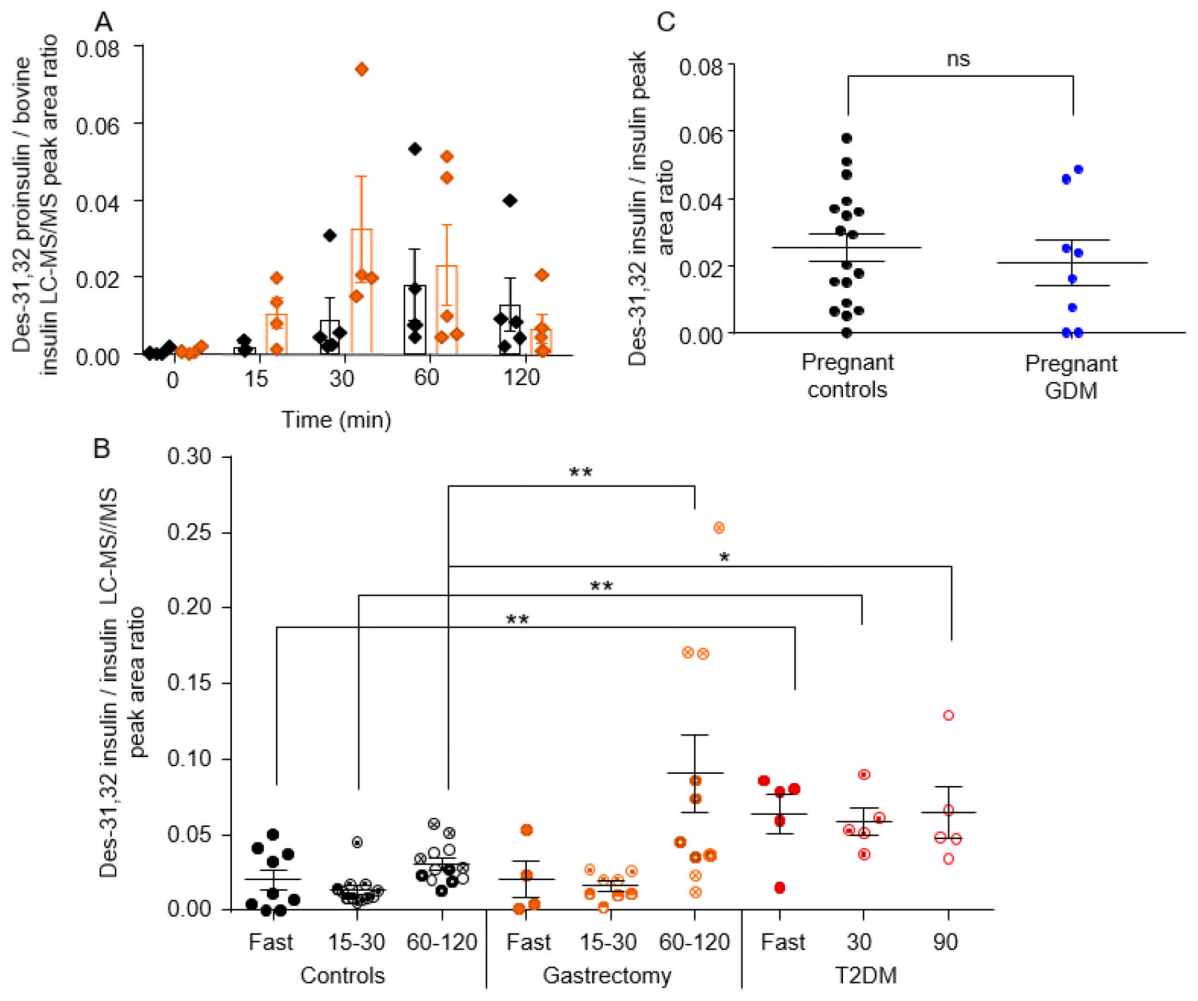
Des 31,32 proinsulin:insulin ratios in different patient groups. A. Mean (±SEM error bars) peak area ratios of des-31,32 proinsulin to bovine insulin internal standard (IS), measured in plasma samples from healthy control (black symbols), and post-gastrectomy patients (orange) after a 50 g oral glucose tolerance test. B. Comparison of des-31,32 proinsulin / insulin ratio in fasted and post OGTT samples from control (black), gastrectomy patients (orange) and patients with T2DM (red), in the fasting state or at the indicated times intervals after glucose ingestion. 15 min (half filled shape), 30 min (open shape with centre dot), 60 min (open shape with thick border), 90 min (open shape) and 120 min (open shape with cross). C. Comparison of des 31,32 proinsulin and insulin chromatography peak area ratio (against bovine insulin) responses in pregnant control (black) and pregnant patients with GDM (blue) at random (non-fasting) sampling timepoints

Due to the limitations in reference standard availability, proinsulin and des-31,32 proinsulin were monitored semi quantitatively with the chromatography peak area values normalised in samples by bovine insulin internal standard and quantified insulin values. Intact proinsulin was detectable in T2DM samples, as previously reported [11], but was only found in a small proportion of control and post-gastrectomy patient samples, so was not analysed further here. Previous analysis of T2DM and associated control samples showed that des-31,32 proinsulin increased after a glucose challenge and that ratios of des-31,32 proinsulin to insulin were higher in T2DM than healthy controls [11]. In the samples from the gastrectomy study, a rise in the plasma des-31,32 proinsulin level was evident after glucose ingestion (Figure 5A), but the overall area under the curve was not significantly different between the gastrectomy and control groups (120 min AUC: control, 2.05 ± 0.58 AU*min, gastrectomy 3.36 ± 0.71 AU*min, p = 0.15).

We were interested in assessing whether patients with T2DM or post-gastrectomy secreted a different ratio of des-31,32 proinsulin to insulin after glucose ingestion, speculating that the T2DM group might have beta cells damaged by chronic hyperglycaemia and hyperstimulation, compared with the gastrectomy group who only exhibit beta cell hypersecretion in the post-prandial state and have largely normal glucose levels. In view of the potential different clearance rates of insulin and des-31,32 proinsulin, we divided the samples according to whether they were fasting, early (15-30 min) or late (60-120 min) during the OGTT (Figure 5B). Ratios of des-31,32 proinsulin to insulin were higher at all time points in the T2DM group compared with controls. In the gastrectomy study, the ratio was no different in patients vs controls in the fasting state or early after the OGTT, but increased at the later time points in the gastrectomy group. From the time courses of insulin and des-31,32 proinsulin shown in figures 4A and 5A, it seems likely that the late increase in ratio arises because insulin levels dropped faster than des-31,32 proinsulin.

As a pilot study, we also analysed insulin and des-31,32 proinsulin in pregnant women undergoing screening for GDM. Plasma samples were collected during routine screening visits when the participants were not fasting. No obvious differences in the des-31,32 proinsulin to insulin ratio were detectable between the pregnant women who screened positive or negative for GDM (Figure 5C), or between pregnant women and the healthy non-pregnant control subjects described above (Figure 5B).

## Discussion

Mass spectrometry is a useful tool to monitor and quantify multiple peptides in a single analysis of a biological sample. The method described here can provide quantitative measurements of insulin, insulin analogues and C-peptide, as well as semi-quantitative information on the presence of proinsulin and its processing intermediates. Synthesis of reference materials and the inclusion of calibration standards for the proinsulin intermediates mean the method could be improved for quantitation of these additional peptides, if required. Still, the current method has the ability to distinguish different forms of insulin and proinsulin simultaneously in a high throughput method and provides substantially more information than a typical immunoassay. Whilst the method is capable of simultaneously quantifying both insulin and C-peptide (albeit with some negative bias in the insulin values in the presented data), the use of LC-MS/MS for routine insulin quantitation is unlikely to replace existing immunoassays. This approach is likely to be applicable for studying patients with suspected insulin secretion disorders or even deficiencies in the activities of processing enzymes such as PC1/3 or PC2, in whom immunoassays yield inaccurate results due to antibody cross-reactivities.

Whilst there has been some development in generating antibodies that span the B-C and C-A chain junctions of proinsulin, potentially enabling the identification of proinsulin species that have not been cleaved at these positions [35], there is substantial complexity in using antibody combinations to distinguish and quantify the multiple partially-processed forms of proinsulin. Different insulin analogues used to treat diabetes are also difficult to distinguish by immunoassay, due to antibody cross-reactivity. The LC-MS/MS method, by contrast, provides rapid and accurate data about a range of proinsulin-derived peptides and insulin analogues from a single analytical run.

Previous reports have shown that insulin secretion in people with T2DM is accompanied by higher proinsulin:insulin release than in healthy individuals [36], perhaps reflecting a state of stressed pancreatic beta cells. An outdated study suggested that up to 50% of circulating immunoreactive insulin in T2DM may be due to proinsulin and des-31,32 proinsulin [2] but without accurate quantification this remains to be confirmed. It is also not clear whether elevated proinsulin or des-31,32 proinsulin defines a subset of people with T2DM, or whether it is indicative or predictive of disease severity or progression. Consistent with previous data our results show elevated des-31,32 proinsulin to insulin ratios in people with T2DM [11], but this was not observed in the fasting state or at early time points after an OGTT in our group of non-diabetic post-gastrectomy patients who hyper-secrete insulin as a consequence of increased GLP-1 levels arising from the altered surgical anatomy [34]. In the gastrectomy group, there was, however, a notable increase in the ratio of des-31,32 proinsulin to insulin at later time points after the OGTT. In T2DM, elevated levels of proinsulin and des-31,32 proinsulin relative to plasma insulin could reflect an increased release of immature insulin vesicles, for instance due to chronic beta cell stimulation and hyperglycaemia. Although we speculated that post-prandial beta cell hyper-secretion in gastrectomy patients could similarly recruit immature secretory granules, this was not evident from the des-31,32 proinsulin to insulin ratios early after glucose ingestion. It seems more likely that the late increased ratio in these patients is an issue of pharmacokinetics, arising from rapidly falling insulin concentrations in the face of slower des-31,32 proinsulin clearance.

Gestational diabetes affects around one in six pregnancies, with prevalence rising in line with population increases in obesity [37]. Pregnancy is associated with increased insulin requirements [38] and GDM arises when insulin secretion is inadequate to meet the higher demand. In this pilot study, des-31,32 proinsulin was detected in the majority of GDM samples, but there were no clear differences compared with non-diabetic pregnant women or our non-pregnant control groups. Further studies will be required to determine whether impaired proinsulin processing is associated with any altered outcomes in pregnancy [39, 40].

## Conclusion

An LC-MS/MS method suitable for the identification and measurement of intact and partially processed proinsulin-derived peptides and therapeutic insulin analogues, was developed and used to analyse plasma samples from study groups with different metabolic conditions. Due to high running costs and longer processing times, mass spectrometry is unlikely to replace existing routine immunoassays for insulin and C-peptide. However, in cases of patients with suspected insulin processing defects, inappropriate use of insulin analogue medication, or where the characterisation of circulatory insulin-related molecules is important, then LC-MS/MS becomes a relevant and highly informative analytical approach. LC-MS/MS can readily differentiate between C-peptide, proinsulin, des-31,32 proinsulin and des-64,65 proinsulin, which cross-react on many standard immunoassays. Whether ratios of proinsulin intermediates to fully processed insulin might be indicative of diabetes subsets, severity or prognosis is an open question for precision approaches to diabetes diagnosis and management, which we hope the newly established method will help to address.

## Supplementary Material

Supplementary Material

## Figures and Tables

**Table 1:** LC-MS parameters for insulin-related peptides

Analyte	Precursor ion (m/z)	Product ion (m/z)	Collision Energy (eV)	Approximate Retention Time (minutes)
Insulin	1162.2	226.3	35	8.58
C-peptide	1007.4	927.5	30	8.66
Proinsulin	1342.1	219.1	35	8.45
Des-31,32 proinsulin	1300.0	785.4	25	8.60
Bovine insulin (Internal standard)	956.3	1120.8	22	8.45
Lispro (Humalog)	1162.2	216.9	40	8.60
Aspart	971.8	661.0	45	8.55
Glargine	867.0	219.1	25	8.26
Detemir	1184	1180.7	20	11.65

**Table 2:** Precision and accuracy for insulin

**Total (Spiked & Endogenous) Concentration (pg/mL) (calculated by Standard Addition)**	182	407	2657	7657
Mean	200	393	1908	5488
Standard Deviation	15	49	115	423
%CV	7.5	12.6	6.0	7.7
%RE	9.8	-3.5	-28.2	-28.3

**Table 3:** Precision and accuracy for C-peptide

**Total (Spiked and Endogenous) Concentration (pg/mL) (calculated by Standard Addition)**	1110	1335	3585	8585
Mean	1050	1214	2885	7406
Standard Deviation	76	101	212	1757
%CV	7.2	8.3	7.3	23.7
%RE	-5.4	-9.1	-19.5	-13.7
