# Supplementary material for: LC-MS/MS based detection of circulating proinsulin derived peptides in patients with altered pancreatic beta cell function
Source: J Chromatogr B Analyt Technol Biomed Life Sci. Author manuscript; Available in PMC 2023 Feb 17. (PMC7614196; doi:10.1016/j.jchromb.2022.123482)
Supplement: Supplementary Material [file EMS164598-supplement-Supplementary_Material.pdf]

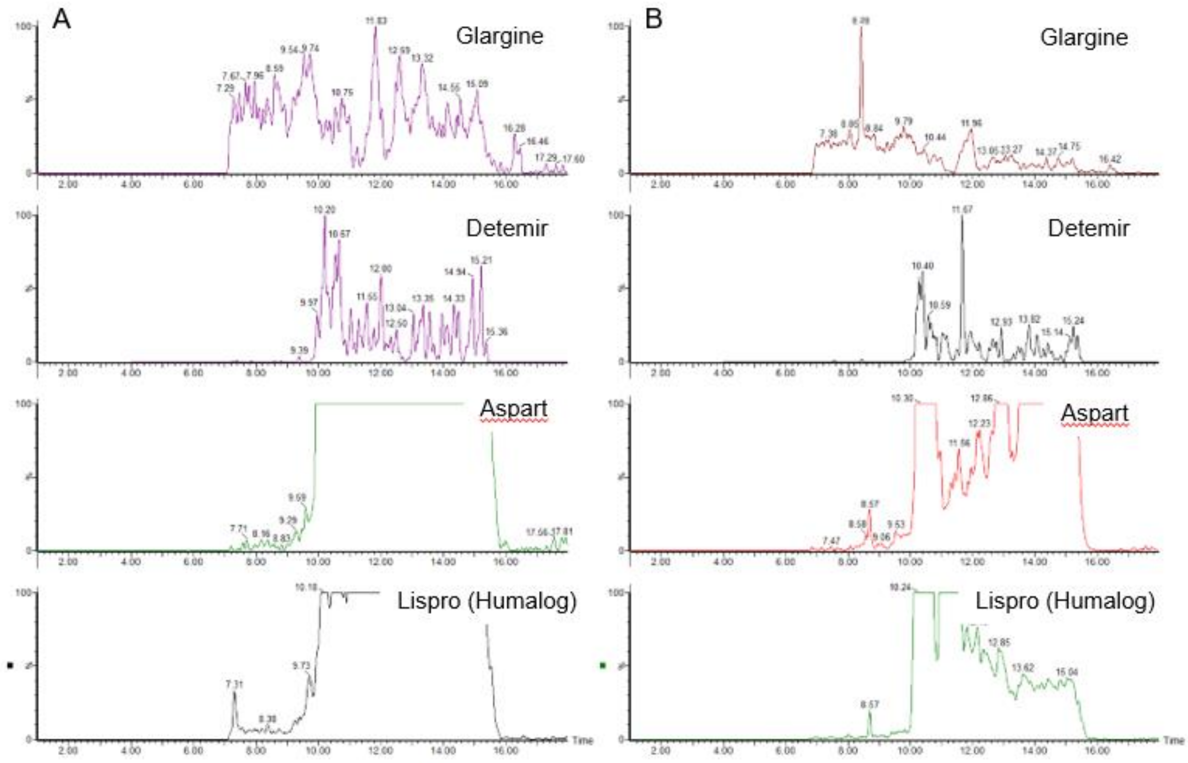

Suppl Figure 1 Chromatograms for all monitored insulin analogue peptide transitions in (A) blank plasma used and (B) 200 pg/mL QC sample.

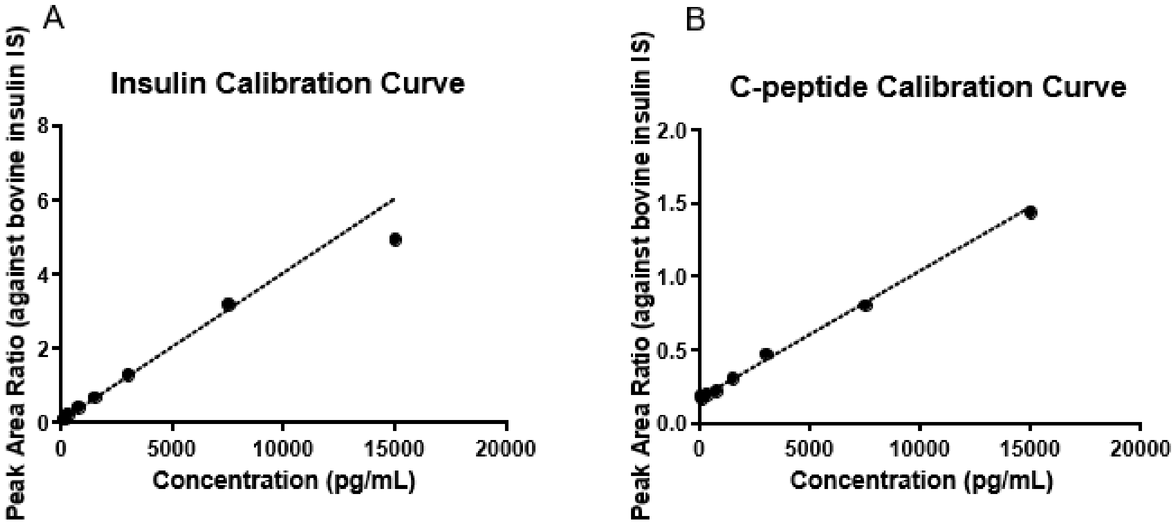

Calibration Curve		Slope	Intercept	R^2 value
Insulin	T2DM samples	0.0003989	0.09014	0.9677
	GDM samples	0.0006100	0.06420	0.9408
	RYGB samples	0.0001474	0.1076	0.8936
C Peptide	T2DM samples	0.0001320	0.1712	0.9902
	GDM samples	0.0005555	0.5027	0.8547
	RYGB samples	0.0001067	0.3197	0.9256

Suppl Figure 2 : Uncorrected calibration curves for (A) insulin and (B) C-peptide, and statistics of least squared (linear) 1/y2 weighted regression for all uncorrected calibration curves used for sample analysis assays

A

Spiked Concentration	Statistics	Analyte	Room temperature (+22°C) for 4 hours	Extraction temperature, on ice (+4°C) for 4 hours	4 x Freeze/Thaw cycles (-70°C/+4°C)	44 days storage (-70°C)
150 pg/mL	Precision (%CV)	Insulin	26.9	13.8	20.7	21.9
		C-peptide	24.1	14.1	7.6	26.2
	Accuracy (%RE)	Insulin	9.2	24.5	-9.6	28.4
		C-peptide	-7.9	-15.7	-19.2	18.6
7500 pg/mL	Precision (%CV)	Insulin	12.5	15.0	7.6	13.2
		C-peptide	14.2	12.4	18.6	17.3
	Accuracy (%RE)	Insulin	13.3	1.6	12.1	-7.0
		C-peptide	10.7	21.0	7.6	18.9

B

Analyte	Precision (%CV)	%Recovery
Insulin	11.8	75.3
C-peptide	11.9	116.0

C

QC Dilution (1:3)	Insulin	C-peptide
%CV	10.4	5.6
%RE	6.1	-17.6

Suppl Table 1: Validation data for insulin and C-peptide quantitation in human plasma QCs, including (A) precision and accuracy values for stability QCs, (B) extraction recovery and (C) precision and accuracy of dilution QCs up to 1:3 ratio.

QC Concentration (pg/mL)	200	600	8000	15000
**Humalog**				
Mean	151.0	509.4	8227.1	14956.2
S.D.	20.4	71.2	1227.5	1930.6
%CV	13.5	14.0	14.9	12.9
%RE	-24.5	-15.1	2.8	-0.3
**Aspart**				
Mean	158.6	492.9	7435.5	13132.6
S.D.	27.2	60.9	965.4	1398.4
%CV	17.2	12.3	13.0	10.6
%RE	-20.7	-17.9	-7.1	-12.4
**Glargine**				
Mean	152.8	534.1	8313.6	15555.6
S.D.	33.7	125.8	1596.2	2018.3
%CV	22.1	23.6	19.2	13.0
%RE	-23.6	-11.0	3.9	3.7
**Detemir**				
Mean	243.0	573.7	9287.2	18291.5
S.D.	46.7	133.8	811.7	2059.9
%CV	19.2	23.3	8.7	11.3
%RE	21.5	-4.4	16.1	21.9

Suppl Table 2: Precision and accuracy values for insulin analogue QCs (n = 6, 4 concentration levels), prepared in blank human plasma and analysed alongside calibration curves.
